# Patient-specific computer modelling – its role in the planning of transcatheter aortic valve implantation

**DOI:** 10.1007/s12471-016-0923-6

**Published:** 2016-11-25

**Authors:** N. El Faquir, B. Ren, N. M. Van Mieghem, J. Bosmans, P. P. de Jaegere

**Affiliations:** 1 Department of Cardiology, Thoraxcenter, Erasmus Medical Center, Rotterdam, The Netherlands; 2 Department of Cardiology, University Hospital Antwerp, Antwerp, Belgium

**Keywords:** Aortic valve stenosis, Transcatheter aortic valve implantation, Computer simulation

## Abstract

Transcatheter aortic valve implantation is increasingly used to treat patients with severe aortic stenosis who are at increased risk for surgical aortic valve replacement and is projected to be the preferred treatment modality. As patient selection and operator experience have improved, it is hypothesised that device-host interactions will play a more dominant role in outcome. This, in combination with the increasing number of valve types and sizes, confronts the physician with the dilemma to choose the valve that best fits the individual patient. This necessitates the availability of pre-procedural computer simulation that is based upon the integration of the patient-specific anatomy, the physical and (bio)mechanical properties of the valve and recipient anatomy derived from in-vitro experiments. The objective of this paper is to present such a model and illustrate its potential clinical utility via a few case studies.

## Introduction

Transcatheter aortic valve implantation (TAVI) is increasingly used to treat patients with severe aortic stenosis who are at increased risk for surgical aortic valve replacement (SAVR) and is projected to be the preferred treatment modality in patients who are at intermediate and supposedly low risk [1–4]. Similar to any other treatment that consists of the implantation of a device into the human body or circulation, outcome depends on specific device-host related factors in addition to patient- and procedure/operator-related variables [5]. Device-host interactions involve the interactions between the patient and device that invariably occur independent of operator-related ones and which affect valve configuration immediately after delivery and, therefore, function and ultimately clinical outcome.

Device-host interactions may in particular play a role in valve performance and outcome after TAVI since – at variance with SAVR – the calcium at the base of aortic root including leaflets is not excised. Therefore, incomplete and/or non-uniform expansion of the frame can occur that in turn may lead to paravalvular leakage (PVL) or a residual gradient [5–8]. Also, the frame extends into the left ventricular outflow tract (LVOT) and may, depending on the depth of implantation in combination with sizing, induce a varying degree of contact stress on the LVOT that in turn may contribute to the occurrence of conduction disturbances [9]. Both conditions are of clinical importance since, depending on the patient’s baseline risk, they may be associated with impaired prognosis [10–12]. Novel generation devices have to a large extent addressed the issue of PVL but are associated with a higher incidence of conduction abnormalities in comparison to preceding valve designs [13, 14]. Yet, PVL still occurs and a number of other (rare) complications can happen such as aortic root rupture, coronary obstruction or valve embolisation [15–18].

On one hand, there is a substantial increase in experience with TAVI that in turn has improved outcome [19, 20]. On the other, the number of different types and sizes of valve technologies increase as well [21]. It is therefore conceivable that device-host interactions will play a dominant role in clinical outcome and that, therefore, the selection of the valve that best fits the individual patient will play a more important role in the further improvement of outcome of TAVI. Such a patient-specific approach – which is endorsed by society and health care policy makers – necessitates the availability of pre-procedural computer simulation that is based upon the integration of the patient-specific anatomy, the physical and (bio)mechanical properties of the valve and recipient anatomy derived from in-vitro experiments [22–24]. The objective of this paper is to present such a model and illustrate its potential clinical utility via a few case studies.

## Methods and results

### Computer simulation with TAVIguide – concept and workflow

#### Concept

Simulation of the implantation of a device into the human body implies the integration of both the physical dimensions and properties of the device (i. e. material) and host (i. e. tissue). The dimensions of device and host are easy to collect (technical information on file, 3D imaging). This also holds for the mechanical properties of the device (mechanical testing) but not for the tissue properties of the patient. In the TAVIguide framework, these tissue properties have been calibrated during initial clinical evaluation studies by using pre- and post-TAVI multislice computed tomography (MSCT) images [25]. The following workflow is (to be) followed:

#### Patient anatomy

**Fig. 1:**
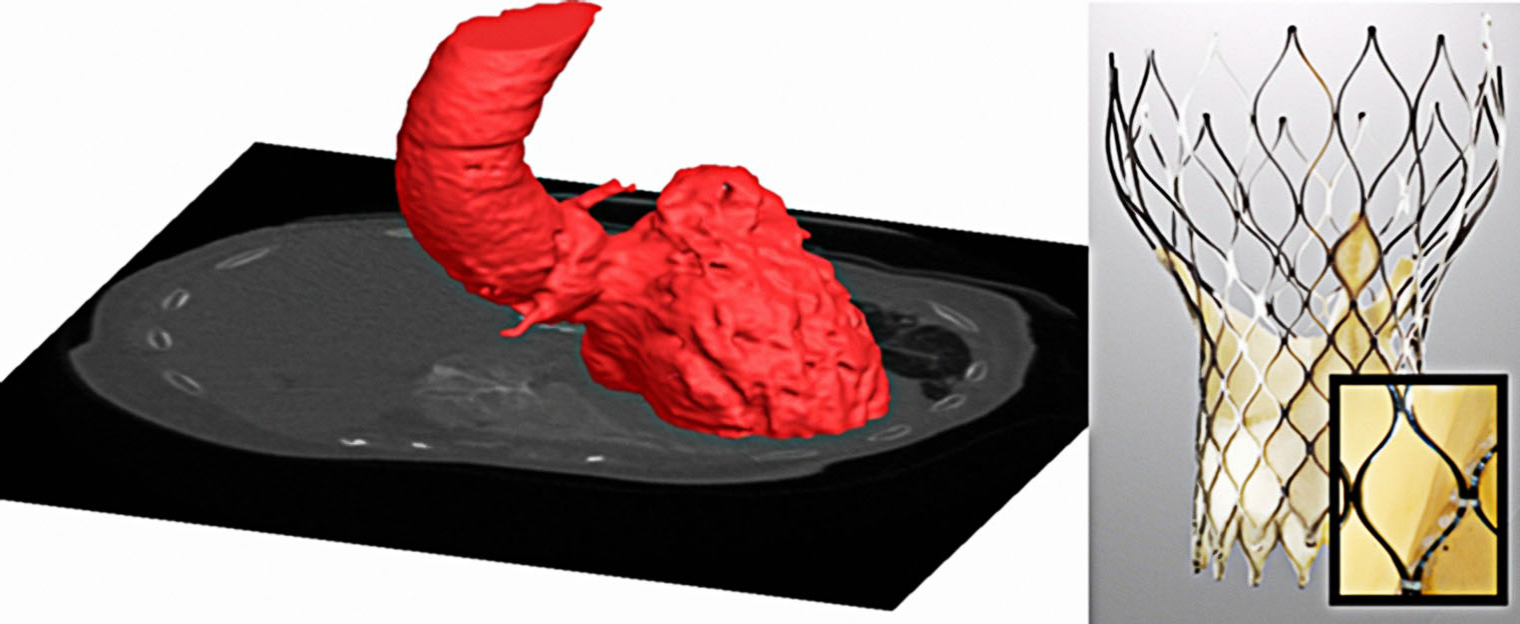
MSCT and 3D reconstruction aortic root and the Medtronic CoreValve with microCT

MSCT is used to obtain geometric and quantitative information on the aortic root using a dedicated scanning and analysis protocol that will be used for 3D reconstruction of the aortic root for subsequent simulation (Fig. 1).

#### Device

As valves are implanted virtually, finite element computer models of valve frames are first developed based upon physical dimensions using microCT, microscopic measurements (resolution of 30 micron, Fig. 1) and mechanical properties. For the latter, in-vitro radial compression tests at body temperature are performed during which the diameter of the frame is reduced over its full length by segmental compression mechanism while recording radial force (RX650, Machine Solutions, Flagstaff, US).

#### Computer modelling

Patient-specific 3D computer models of the aortic root including the calcified native leaflets are reconstructed using MSCT and image segmentation techniques (Mimics Software, Materialise, Leuven, Belgium). Varying mechanical properties are automatically assigned to different tissue regions within the LVOT-aortic root complex. The biomechanical properties used in the TAVIguide simulations are improved by calibration and validation studies [25]. The computer generated valve frames are then implanted virtually into the patient’s specific anatomy using finite element computer simulations using the Abaqus/Explicit finite element solver (Dassault Systèmes, Paris, France).

### Clinical validation

**Fig. 2:**
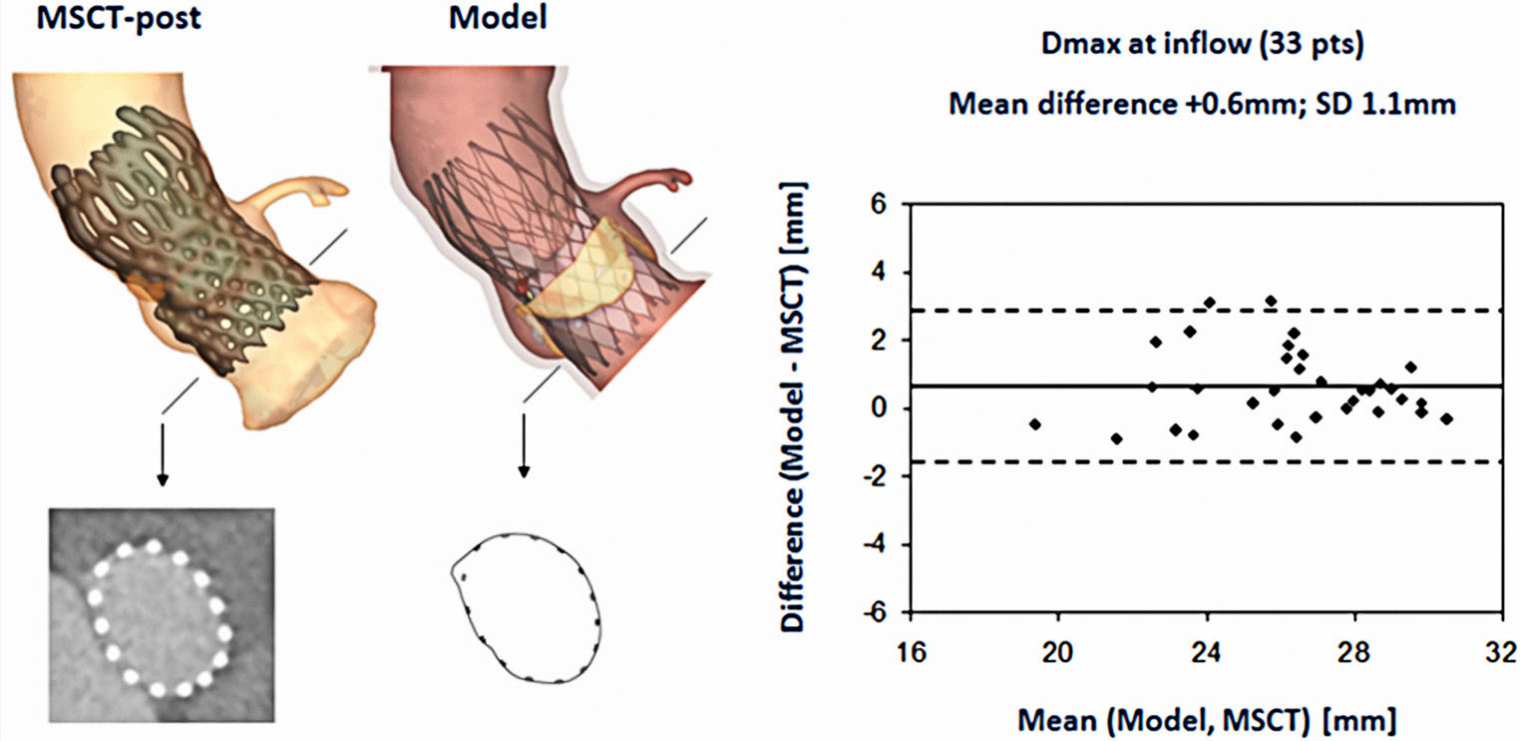
Observed (MSCT post-TAVI) and predicted (computer simulation) valve geometry with correlation between MSCT and predicted maximal diameter (Dmax) [25]

**Fig. 3:**
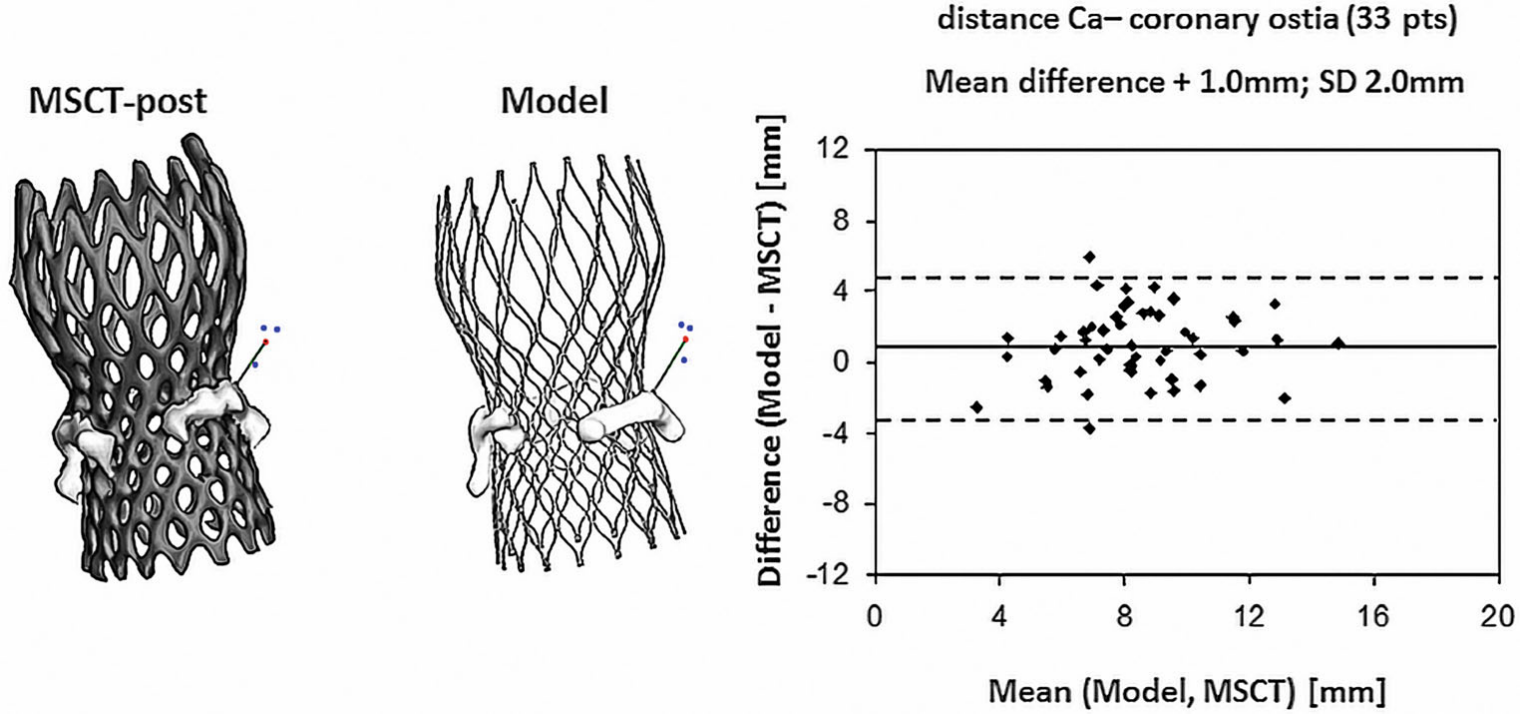
Comparison between MSCT and the predicted distance between the coronary ostia and the closest calcium nodule with correlation between MSCT and predicted distance from the coronary ostia to the calcium nodule [25]

Two multicentre observational studies have been conducted for the clinical validation of the TAVIguide software [25, 26]. The first sought to assess the accuracy of the software to predict frame morphology, dimensions and aortic leaflet displacement after valve implantation [25]. Quantitative data of axial frame morphology (minimal diameter (Dmin), maximal diameter (Dmax), cross-sectional area and perimeter) of 33 patients treated with the Medtronic CoreValve System (MCS) and of 6 patients treated with the Edwards Sapien XT (ESV) obtained by MSCT post-TAVI (observed frame morphology & dimensions), were compared with those obtained from the computer model (predicted frame morphology & dimensions) (Fig. 2). Similarly, displacement of the aortic leaflet calcifications, quantified by the distance between the coronary ostia and the closest calcium nodule, was compared between MSCT and model (Fig. 3).

During the simulation, all steps of the clinical TAVI procedure were respected including pre-dilatation, valve size, depth of implantation and post-dilatation if applied. The depth of implantation was matched with the depth of implantation during actual valve implantation by overlaying the 3D aortic root model derived from the software after simulation of valve implantation with the one derived from MSCT post-TAVI followed by evaluating the resulting alignment of the inflow of the valve frame of the 3D model with the one of the MSCT post-TAVI, which was used as reference. Simulations were repeated until correct alignment was obtained, which was used for the validation analysis. For the pre- and (if applicable) post-dilatation, the same size of the balloon that was used during the in-vivo implantation was used during the computer simulation.

Bland-Altman analysis revealed a strong correlation between the observed (MSCT) and predicted frame dimensions although small differences were detected for e. g. Dmin at the inflow (mean ± SD, MSCT vs. model: 21.6 ± 2.4 mm vs. 22.0 ± 2.4 mm; difference ± SD: −0.4 ± 1.3 mm, *p* < 0.05) and Dmax (mean ± SD, 25.6 ± 2.7 mm vs. 26.2 ± 2.7 mm; difference ± SD: −0.6 ± 1.0 mm, *p* < 0.01). An example of the correlation between the observed and predicted Dmax is shown in Fig. 2. The observed and predicted distances from coronary ostia to the closest calcium nodule were highly correlated for the left and right coronary ostia (R^2^ = 0.67 and R^2^ = 0.71, respectively *p* < 0.001) (Fig. 3). This distance was slightly overestimated by the model for both coronary arteries. Dedicated software, thus, allows accurate prediction of frame morphology and calcium displacement after valve implantation, which may help to improve outcome.

**Fig. 4:**
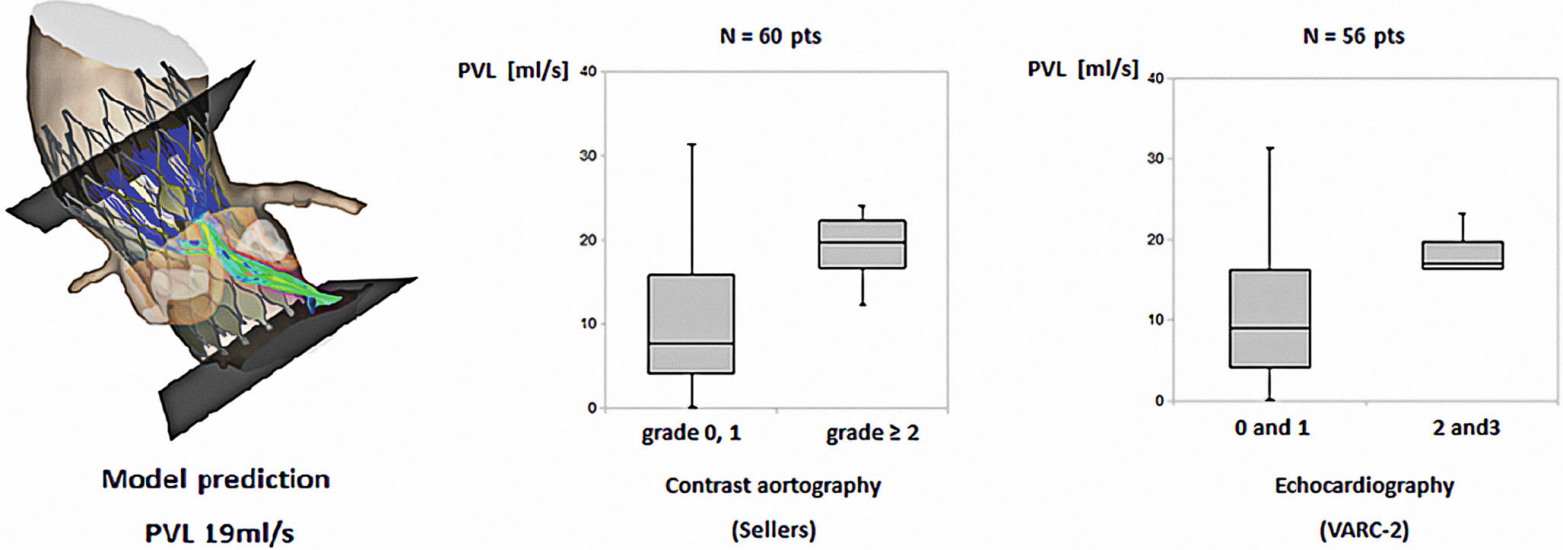
Blood flow domains including PVL channels were derived from predicted frame deformation and box plot analysis from the observed and predicted PVL [26]

The second study focused on the accuracy of the model for the prediction of PVL after TAVI [26]. Similar to the first validation study, pre-operative MSCT was used to generate 3D models of the aortic root of 60 patients treated with a MCS valve. Implantation of virtual valve models was simulated using finite element computer modelling. Blood flow domains including PVL channels were derived from predicted frame and aortic root deformation (Fig. 4). Computational fluid dynamics was used to model blood flow during diastole to assess PVL. Predicted and observed PVL (angiography, echocardiography) were compared. Moderate or more PVL was seen in 15 patients (25%) by angiography (Sellers aortic regurgitation grade ≥2) and in 9 (15%) by echocardiography (short-axis circumferential extent ≥10%, VARC-2). Box plot analysis revealed good agreement between observed and predicted PVL (Fig. 4). ROC analysis indicated 16.25 ml/sec (reference angiography) and 16.0 ml/sec (reference echocardiography) as cut-off values that best differentiated patients with none-to-mild and moderate-to-severe PVL. Sensitivity, specificity, positive predictive value, negative predictive value and accuracy were 0.80, 0.80, 0.57, 0.92 and 0.80, respectively (reference angiography) and were 0.72, 0.78, 0.35, 0.94 and 0.73 (reference echocardiography).

### Case studies

**Fig. 5:**
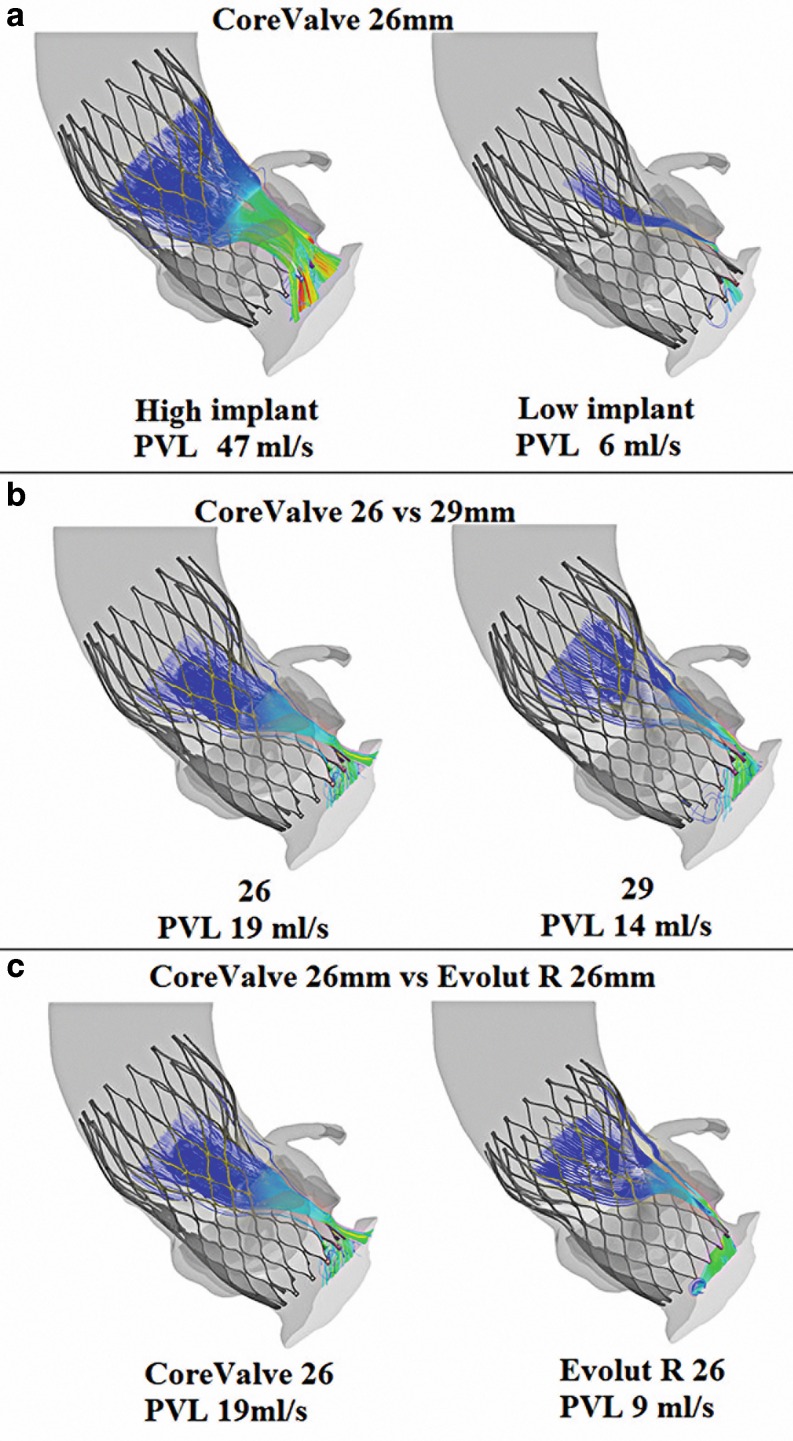
**a** change in depth of implantation; lower implant resulted in less PVL. **b** change in valve size; a larger valve resulted in a reduction of PVL. **c** change in valve type without changing size and implantation depth: significant less PVL after Evolut R implantation

The clinical role and potential of computer simulation is illustrated by case examples in which depth of implantation, valve size or valve type have been changed while using the same baseline anatomy (i. e. MSCT patient) to assess the effect of those changes on PVL (Fig. 5). Implanting a 26 mm MCS in a lower position resulted in a 87% reduction in PVL. Using the same implantation depth but a 29 instead of a 26 mm valve resulted in a 26% reduction in PVL. Changing valve type while maintaining similar implantation depth and valve size did have an effect of 53% reduction in PVL when using the Evolut R valve instead of the MCS.

## Discussion

Outcome of a medical intervention such as TAVI depends on a combination of patient-, procedure- or operator- and device-related factors, each of which contributes to outcome in a different way and magnitude. As the outcome of TAVI improves due to improved patient selection and operator performance, it is hypothesised that the interaction between device and host will play a more dominant role. Obviously, valve technology changes and improves as well, yet clinical issues will remain as a prosthesis is implanted in a very specific biological environment that necessitates a patient-customised valve that unfortunately does not exist. In clinical practice, however, the physician has the choice between an increasing number of valve types and sizes that in turn confronts him/her with the responsibility to choose the valve that best fits the individual patient. To support the physician in this process, simulation of valve implantation such as the one that is described here is a novel and reachable step forward.

**Figure:**
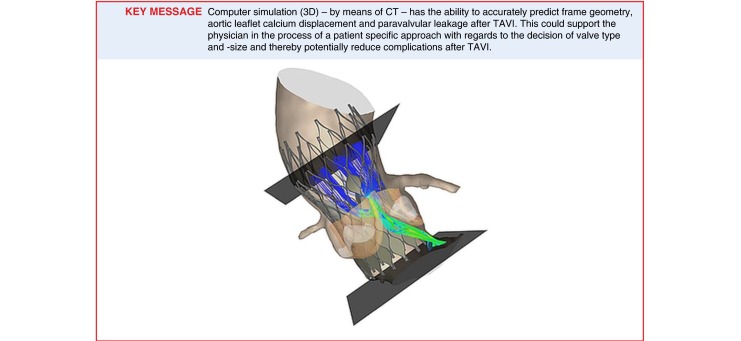

The simulation presented above has shown in a selected series of patients and centres that computer simulation with the TAVIguide accurately predicts frame geometry, aortic leaflet calcium displacement and, thus, risk of coronary occlusion in addition to PVL directly after MCS and ESV implantation [25, 26]. Also, the versatility of the program is illustrated with the case studies in which the effect of changing either the depth of implantation, valve size or type in the same patient on the severity of PVL is shown.

As mentioned, the information stems from a selected series of patients in centres that perform high-quality MSCT in all their patients referred for TAVI. It remains to be seen whether similar correlations as reported in the two pilot observational studies will be observed when offering computer simulation to a wider range of patients and centres with varying degrees of access to MSCT and MSCT image quality. Also, the current computer simulation program only offers simulation for a limited number of valve types. This implies that the development of software for computer simulation is a continuous process in which novel valve technologies have to be incorporated into the software algorithm. In addition, as novel generation valves appear to have addressed the issue of PVL, other interactions – conduction abnormalities in particular – need to be predicted by the simulation program. For instance, the incidence of moderate or severe aortic regurgitation after Lotus and Edwards Sapien 3 valve implantation is 1% but are associated with a new pacemaker implantation rate of 32 and 17%, respectively [13, 14].

In addition to the incorporation of all clinically available valve technologies and the capacity to predict all clinically relevant (i. e. frequency and severity) device-host interactions, the clinical role of the computer simulation program needs to be further established by appropriately designed prospective and ultimately randomised clinical trials in a wide segment of patients scheduled for TAVI.

The development of patient-specific treatment and treatment planning (patient-tailored approach) is strongly recommended by the health care authorities and is also more and more embraced by the medical community [27]. More specifically, to evaluate its effectiveness in clinical practice, a European multicentre study is currently being designed. The multicentre character reflects the interest of the medical community in this program. With respect to logistics, time and costs, only a MSCT scan needs to be uploaded via a web-based system that is followed by simulation with a written report within 24–48 h. Costs are not defined yet and will ultimately depend on the ratio of input, volume and eventual benefit.
